# Analysis of miRNAs in the Heads of Different Castes of the Bumblebee *Bombus lantschouensis* (Hymenoptera: Apidae)

**DOI:** 10.3390/insects10100349

**Published:** 2019-10-16

**Authors:** Meijuan Liu, Jiaxing Huang, Guangshuo Zhang, Xiaofeng Liu, Jiandong An

**Affiliations:** 1 Key Laboratory for Insect-Pollinator Biology of the Ministry of Agriculture and Rural Affairs, Institute of Apicultural Research, Chinese Academy of Agricultural Sciences, Beijing 100093, China; liumeijuan0711@163.com (M.L.); huangjiaxing@caas.cn (J.H.); zhangguangshuo77@126.com (G.Z.); 2 School of Life Science, Peking University, Beijing 100871, China; evolution@pku.edu.cn

**Keywords:** pollinators, *Bombus lantschouensis*, microRNA, Solexa sequencing

## Abstract

Bumblebees are important insect pollinators for many wildflowers and crops. MicroRNAs (miRNAs) are endogenous non-coding small RNAs that regulate different biological functions in insects. In this study, the miRNAs in the heads of the three castes of the bumblebee *Bombus lantschouensis* were identified and characterized by small RNA deep sequencing. The significant differences in the expression of miRNAs and their target genes were analyzed. The results showed that the length of the small RNA reads from males, queens, and workers was distributed between 18 and 30 nt, with a peak at 22 nt. A total of 364 known and 89 novel miRNAs were identified from the heads of the three castes. The eight miRNAs with the highest expressed levels in males, queens, and workers were identical, although the order of these miRNAs based on expression differed. The male vs. queen, male vs. worker, and worker vs. queen comparisons identified nine, fourteen, and four miRNAs with significant differences in expression, respectively. The different castes were clustered based on the differentially expressed miRNAs (DE miRNAs), and the expression levels of the DE miRNAs obtained by RT-qPCR were consistent with the read counts obtained through Solexa sequencing. The putative target genes of these DE miRNAs were enriched in 29 Gene Ontology (GO) terms, and catalytic activity was the most enriched GO term, as demonstrated by its association with 2837 target genes in the male vs. queen comparison, 3535 target genes in the male vs. worker comparison, and 2185 target genes in the worker vs. queen comparison. This study highlights the characteristics of the miRNAs in the three *B. lantschouensis* castes and will aid further studies on the functions of miRNAs in bumblebees.

## 1. Introduction

As important pollinators of wildflowers and crops, bumblebees play a significant role in natural and agricultural ecosystems [1,2,3,4]. Since 1987, bumblebees have been used for pollination in commercial production, which has greatly promoted the development of artificial rearing technology [3]. Bumblebees are social insects with three castes—males, queens, and workers. During initial colony formation, the queen collects nectar and pollen and lays eggs. After the first batch of workers has hatched, the queen focuses on laying eggs. The main functions of the workers are gathering food, secreting wax, nesting, feeding larvae, and cleaning. Males are colony philanderers and hatch from unfertilized eggs, and their function is to mate with new queens [5]. Therefore, the functions of the three castes in bumblebee colonies are significantly different. MicroRNAs (miRNAs) are molecules that play regulatory roles in biological functions, and an exploration of the miRNAs expressed in the three castes will be helpful for understanding their molecular regulatory function in bumblebees.

miRNAs are endogenous, single-stranded, and non-coding small RNAs approximately 22 nt in length that induce translational inhibition or target gene degradation by binding to complementary mRNA sequences. These miRNAs are ubiquitous in eukaryotes and are highly conserved in related species [6,7]. It has been demonstrated that miRNAs play regulatory roles in many biological processes in animals, including embryonic development, tissue differentiation, cell proliferation, apoptosis, and morphogenesis [8,9]. Previous studies have shown that miRNAs play important roles in regulating neuronal differentiation, synaptic plasticity, and behavioral regulation in mammalian brains. Since the head of the honeybee is the control center for learning, memory, and cognition [10,11], research on the miRNAs expressed in the honeybee head can reveal functions related to behavior.

In honeybees, the expression levels of miRNAs are related to diverse biological functions, such as caste differentiation, foraging and dancing behaviors, and ovary activation [12,13,14], and eight miRNAs expressed in the head of the *Bombus lantschouensis* queen regulate oviposition [15]. Furthermore, the expression pattern of miR-315 was measured to validate the stability of the best miRNA candidate gene in the bumblebee queen in response to reproduction [16]. In *Apis* and *Drosophila*, six miRNAs show differential expression between the queen and worker larvae, and the target genes of these miRNAs are involved in development and reproductive differentiation [17]. However, the characteristics of the miRNAs expressed in the three bumblebee castes have not been explored, and their functions in the different bumblebee castes remain unknown.

The European bumblebee, *Bombus terrestris*, is the commercial species most commonly used for agriculture pollination worldwide, but strong invasion of *B. terrestris* has caused a decline in local bumblebees in many parts of the world, which has raised concerns regarding the conservation of local bees [18,19,20,21]. Recent studies have shown that the habitats of Chinese bumblebees are also threatened by the invasive species *B. terrestris* [22]. Therefore, further studies on the rearing of local bumblebees for pollination purposes is crucial for avoiding invasion of the introduced species. *B. lantschouensis* is one of the most important local bumblebee species used commercially in China due to its beneficial characteristics of easy rearing in captivity, large colony size, large number of queens, high mating rate, and pollination services [23,24].

In the present study, the miRNAs expressed in the three castes of *B. lantschouensis* were identified and characterized based on high-throughput sequencing, and the significant differences in the expression levels of these miRNAs among the castes were validated by RT-qPCR. The target genes of the miRNAs were predicted, and their regulatory pathways were analyzed. Our results will enhance the basic knowledge of miRNAs expressed in different bumblebee castes and might help clarify their important biological functions in bumblebees and thus aid in the rearing of bumblebees for agricultural pollination.

## 2. Materials and Methods

### 2.1. Samples

*Bombus lantschouensis* samples were collected from the bumblebee rearing room at a laboratory in the Institute of Apicultural Research, Chinese Academy of Agricultural Sciences, Beijing, China. All the colonies were fed sugar syrup (50% sugar content, w/vol) and the same pollen pellets (apricot:oilseed rape = 1:1, mixture of fresh pollen with pure water) every other day to prevent fermentation of the food under the rearing conditions (temperature of 29 °C ± 0.5 °C, 55% ± 5% relative humidity, and continuous darkness). The pollen pellets were collected from honeybees (*Apis mellifera*) using pollen traps [23]. One week after emergence, whole bodies of the males, queens, and workers were placed directly into liquid nitrogen. The heads were cut and collected in Eppendorf tubes independently. Three biological replicates of each caste were included in the study. In total, nine head samples, including three males, three queens, and three worker heads, were collected.

### 2.2. RNA Isolation, Library Construction, and Sequencing

One head was considered as one sample, and a total of nine bumblebee heads were used in this study. Total RNA of each sample was isolated using the TRIzol reagent (Invitrogen, Carlsbad, CA, USA). After the homogeneity of the sample was confirmed, each head was digested with 1 mL of the TRIzol reagent and incubated for 5 min at room temperature. Then, 0.2 mL of chloroform was added to each sample, and the mixture was incubated for 3 min. The sample was centrifuged for 15 min at 12,000× *g* and 4 °C. The aqueous phase was added to 0.5 mL of isopropanol, and the mixture was incubated for 10 min and centrifuged for 10 min at 12,000× *g* and 4 °C. The pellet was resuspended in 1 mL of 75% ethanol and vacuum dried, and these steps were performed twice. The pellet was then resuspended in 50 μL of RNase-free water. The RNA integrity and quantity were determined by 1% agarose gel electrophoresis and using a Micro-Volume UV–Vis spectrophotometer (NanoDrop-2000, Waltham, MA, USA). For construction of the small RNA library, total RNA was separated by 15% denaturing polyacrylamide gel electrophoresis, and the 18–30 nt fragments were purified. The gel-purified small RNAs were then ligated to a 5’ adapter and a 3’ adapter using T4 RNA ligase. The ligated fragments were reverse-transcribed and amplified by PCR. The purified PCR products were then sent to Berry Genomics Co., Ltd. (Beijing, China) for high-throughput sequencing on the Solexa high-throughput platform.

### 2.3. Read Filtering and Mapping

The raw data were assessed using NGS QC Toolkit (version 2.3), and Cutadapt (version 1.16) was used to discard reads with more than two N bases, 5’ and 3’ adapters from the reads, reads with more than 10 poly-A sequences, low-quality reads containing a 5’ end with a cutoff of 15 and a 3’ end with a cutoff of 10, and reads with a length less than 18 nt and more than 30 nt. All clean reads were mapped to the *B. terrestris* genome using Bowtie (version 1.1.1) [25], and only reads with no more than two mismatch sequences were screened.

### 2.4. Prediction of miRNAs

Known and novel miRNAs were predicted using miRDeep2 with the default parameters [26]. Specifically, miRNAs were predicted from each dataset of small RNA clean reads. All predicted miRNAs with scores less than 4 were removed, and the mapped small RNA clean reads were aligned to the mature miRNAs in all species included in miRBase 22.1 [27]. The sequences that exhibited a perfect match in the seed sequence and had no more than two mismatches in other locations were extracted as conserved miRNAs. *B. terrestris* and *B. lantschouensis*, which are species that belong to the same subgenus of *Bombus* s. str., exhibited a close relationship [28]. Since the *B. lantschouensis* genome has not been published, we used the *B. terrestris* genome as the reference genome. The unmatched sequences were aligned to the *B. terrestris* genome. The typical miRNA precursor consists of a 70-nt-long single-strand RNA, which can form a stem-loop structure. The secondary structure of the miRNA precursors were predicted using RNAfold software with the default parameters. The sequences in the bumblebee genome that formed the typical miRNA precursor stem-loop structure were considered to encode novel miRNAs.

### 2.5. Differential Expression Analysis of miRNAs

The miRNA read counts were normalized to transcript per million (TPM) for better visualization and comparability [29]. The DESeq R package [30] was used to analyze the differentially expressed miRNAs (DE miRNAs; Benjamini and Hochberg method corrected *p*-value < 0.05, |log2-fold change (FC)| ≥1) identified from each of the following comparisons: (1) male vs. queen, (2) male vs. worker, and (3) worker vs. queen. A cluster analysis of the DE miRNAs was performed using TBtools (version 0.665) [31].

### 2.6. Target Gene Prediction and Gene Ontology (GO) and Kyoto Encyclopedia of Genes and Genomes (KEGG) Pathway Enrichment Analyses

Due to the fact that information on the *B. lantschouensis* genome is not available, the 3′-untranslated region (3’-UTR) sequences from the *B. terrestris* genome were used to predict the target genes of the DE miRNAs with RNAhybrid (version 2.2.2) [32], miRanda [33], and PITA (version 1.6) [34]. We extracted the overlapping target genes identified using the three software packages, and the biological functions of the predicted target genes were predicted through GO and KEGG pathway analyses using Blast2GO software [35]. The *p*-values of the significantly enriched GO terms and KEGG pathways were obtained using the hypergeometric distribution and Fisher’s exact methods. Only terms with *p* < 0.05 in the secondary classification of the GO terms and KEGG pathways were considered significantly enriched.

### 2.7. miRNA Extraction and RT-qPCR

To validate the predicted miRNA expression levels, miRNAs were extracted from the heads of *B. lantschouensis* males, queens, and workers one week after emergence using the miRcute miRNA Isolation kit (Tiangen, Beijing, China) according to the manufacturer’s recommended protocol. The RNA integrity and quantity were determined by 1% agarose gel electrophoresis. cDNA synthesis was performed using the miRcute Plus miRNA First-Strand cDNA synthesis kit (Tiangen) following the manufacturer’s recommended protocol. RT-qPCR was performed using the miRcute Plus miRNA qPCR kit according to the manufacturer’s instructions, and U6 small non-coding RNA was used as the reference gene in the RT-qPCR analysis [36]. The relative expression of the miRNAs was determined using the comparative quantity (2^−ΔΔCT^) method [37]. In total, 19 DE miRNAs were selected and verified. The poly(A) tail method yielded a lower number of false positives compared with those obtained using the stem-loop primer method. We thus designed forward primers (Table 1) for the various miRNAs using Primer Premier 3 and measured the expression of these miRNAs using the universal reverse primer provided with the miRcute Plus miRNA qPCR kit. Three technical replicates and three biological replicates were used to analyze each caste. The differences in the relative expression levels of the miRNAs obtained in the two-cast comparisons (male vs. queen, male vs. worker, and worker vs. queen) were analyzed using an independent-sample *t*-test with SPSS 20.0 software. Prior to the *t*-test, the normality and homoscedasticity were checked using Shapiro–Wilk and Levene tests. If the data did not exhibit a normal distribution, the differences in miRNA expression levels were tested using the non-parametric Mann–Whitney U method.

## 3. Results

### 3.1. Analysis of Sequencing Data

**Figure 1:**
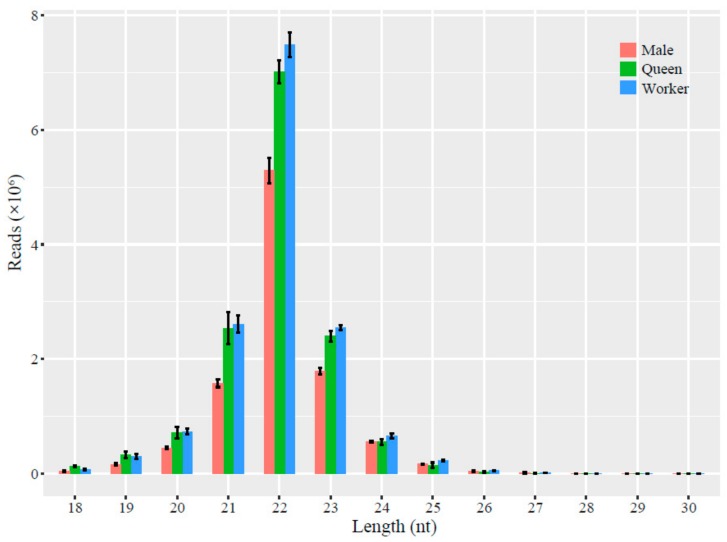
Distribution of the lengths of the small RNA reads in the three *B. lantschouensis* castes. The black vertical lines represent error bars. The size distribution of the 18–30 nt clean reads obtained from all three groups was assessed. Most sequences in the three libraries were 21–23 nt in length, and the most abundant size was 22 nt.

Solexa sequencing yielded 14,326,369, 14,736,596, and 16,317,406 raw reads from the males, queens, and workers; after removal of the short reads (<18 nt), low-quality reads, and reads with adapters, the male, queen, and worker libraries contained 13,569,008, 13,949,908, and 15,413,820 clean reads, respectively (Table 2). Further analysis showed that 71.61%, 69.76%, and 70.33% of the clean reads in the male, queen, and worker libraries were successfully mapped to the *B. terrestris* genome, respectively (Table 2). The length of the small RNA reads in the three castes of *B. lantschouensis* was between 18 nt and 30 nt, with a peak at 22 nt (Figure 1). The percentage of reads with a length of 22 nt in males was 52.08%, whereas the corresponding percentages in queens and workers were 50.59% and 50.64%, respectively.

### 3.2. Identification and Expression of Known and Novel miRNAs

**Figure 2:**
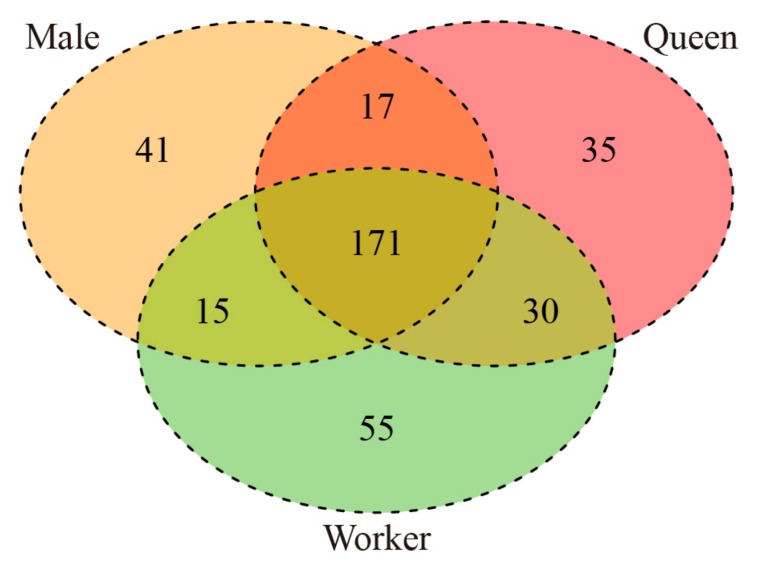
Distribution of known microRNAs (miRNAs) in the three *B. lantschouensis* castes. The Venn diagram displays the distribution of 364 unique known miRNAs among the male, queen, and worker libraries.

The analysis of the three castes of *B. lantschouensis* identified 364 known miRNAs (Figure 2) and 89 novel miRNAs (Supplementary Figure S1). The estimated false positive rate obtained with a score cutoff of four was 0.09 ± 0.01 among the known miRNAs and 0.06 ± 0.01 among the novel miRNAs. In total, 84 of the 364 known miRNAs were aligned to the *Apis mellifera* genome (Supplementary Figure S2), and the 10 unique miRNAs with the highest abundance were extracted (Table 3). Interestingly, eight of the top 10 miRNAs (ame-miR-1-3p, ame-miR-276-3p, ame-miR-8-3p, ame-bantam-3p, ame-miR-2796-3p, ame-miR-317-3p, ame-miR-7-5p, and ame-miR-277-3p) were present in males, queens, and workers, although their rankings showed differences among the castes. The analysis of the different caste libraries revealed that ame-miR-1-3p was the most abundant miRNA with 989,563 reads.

### 3.3. DE miRNAs in Males, Queens, and Workers

**Figure 3:**
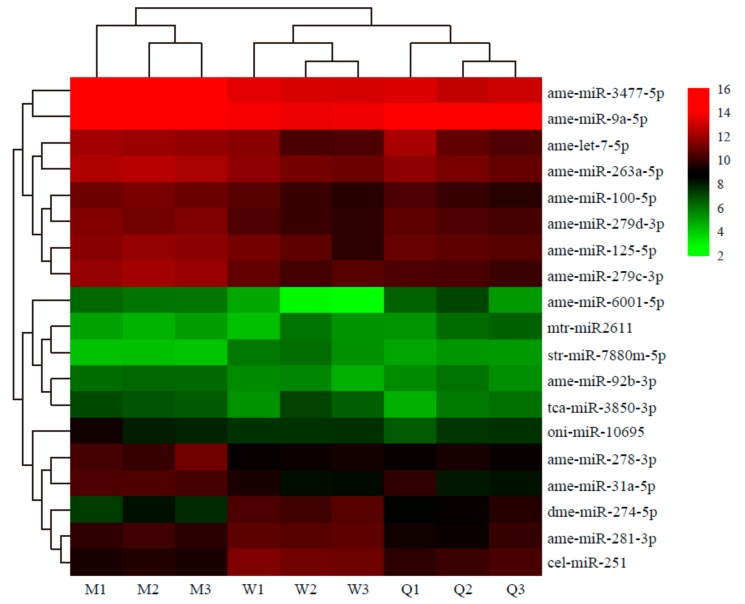
Heat map diagram showing the differentially expressed miRNAs (DE miRNAs) in the three *B. lantschouensis* castes. Nineteen differentially expressed miRNAs with |fold change| ≥1 and *p* < 0.05 were screened using the DESeq R package. The rows represent the different miRNAs, and the columns represent males (M1, M2, M3), workers (W1, W2, W3), and queens (Q1, Q2, Q3). The expression data for each miRNA were calculated from three biological replicates.

In total, 19 miRNAs exhibited significantly different expression among the male, queen, and worker libraries (q < 0.05 and |log2-fold change| >1): 18 miRNAs showed differential expression in the male vs. female comparisons (seven upregulated and two downregulated miRNAs were obtained in the male vs. queen comparison, and 11 upregulated and three downregulated miRNAs were identified in the male vs. worker comparison), and three upregulated miRNAs and one downregulated miRNA were found in the worker vs. queen comparison (Table 4). A greater number of DE miRNAs were obtained in the male vs. female (male vs. queen or male vs. worker) comparisons compared with the worker vs. queen comparison. The clustering analysis suggested that the different castes were significantly grouped based on the DE miRNAs (Figure 3). Among the 19 DE miRNAs identified in this analysis, dme-miR-274-5p was differentially expressed in the male vs. queen, male vs. worker, and worker vs. queen comparisons; ame-miR-263a-5p, ame-miR-278-3p, ame-miR-279c-3p, and ame-miR-3477-5p showed differential expression in both the male vs. queen and male vs. worker comparisons; ame-miR-6001-5p and cel-miR-251 were differentially expressed in both the male vs. worker and worker vs. queen comparisons.

### 3.4. Prediction of Target Genes of DE miRNAs and Functional Enrichment Analysis

**Figure 4:**
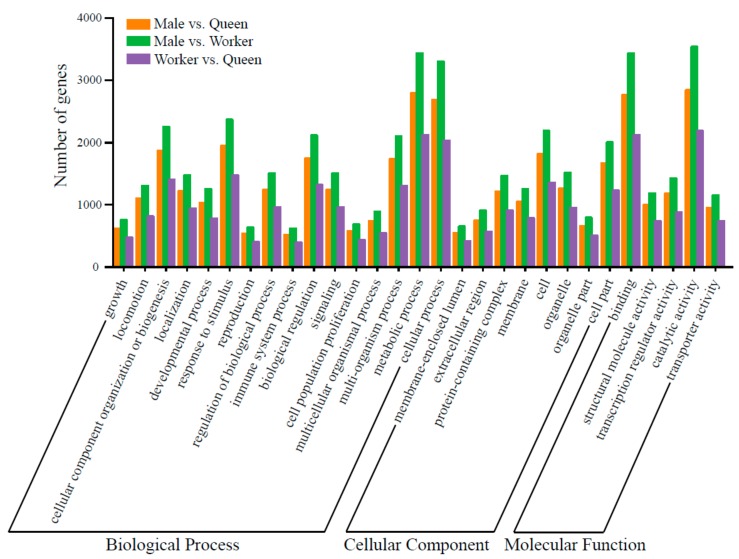
Gene Ontology (GO) enrichment analysis of potential target genes of the DE miRNAs of *B. lantschouensis* obtained in the male vs. queen, male vs. worker, and worker vs. queen comparisons. The x-axis shows the GO category, and the y-axis indicates the number of genes.

**Figure 5:**
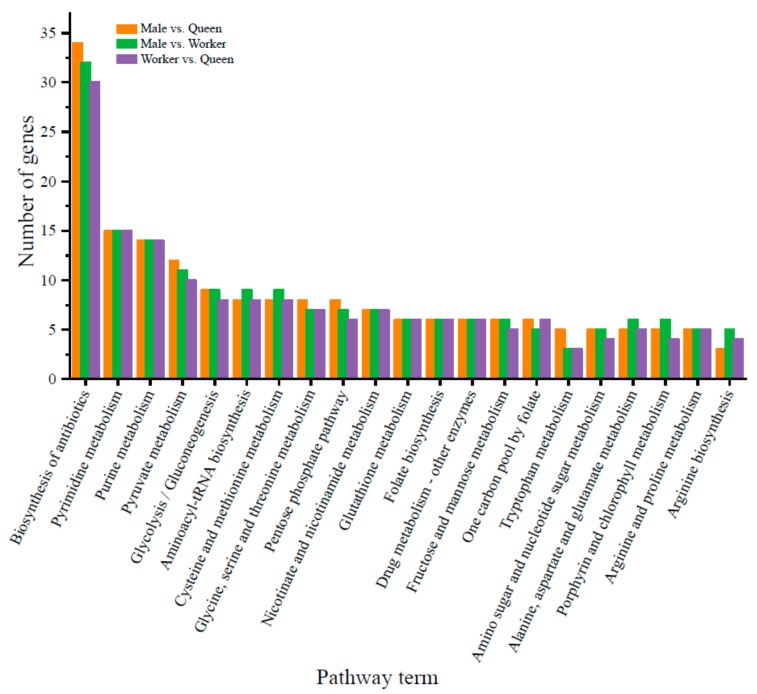
Kyoto Encyclopedia of Genes and Genomes (KEGG) pathways of potential target genes of DE miRNAs of *B. lantschouensis* obtained in the male vs. queen, male vs. worker, and worker vs. queen comparisons. The x-axis shows the pathway terms, and the y-axis indicates the number of genes.

The intersection of the data obtained using RNAhybrid (version 2.2.2), miRanda, and PITA (version 1.6) predicted 3515, 4333, and 2658 target genes for nine miRNAs obtained in the male vs. queen comparison, 14 miRNAs identified in the male vs. worker comparison, and four miRNAs obtained in the worker vs. queen comparison, respectively. The putative target genes of these DE miRNAs obtained in the male vs. queen, male vs. worker, and worker vs. queen comparisons were all enriched in 29 GO terms. Catalytic activity, metabolic process, and binding were the top three most abundant terms (Figure 4). The putative target genes were enriched in 21 KEGG pathways, and the biosynthesis of antibiotics, pyrimidine metabolism, and purine metabolism were the top three most abundant pathways (Figure 5).

### 3.5. Validation of Differential miRNA Expression by RT-qPCR

**Figure 6:**
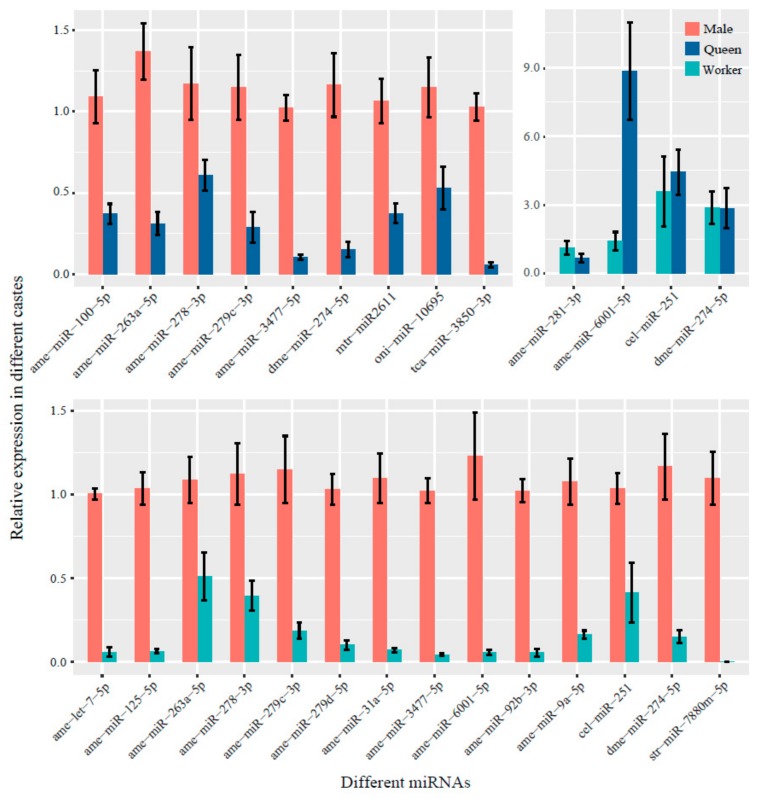
RT-qPCR validation of the DE miRNAs in the three *B. lantschouensis* castes identified by Solexa small RNA sequencing. RT-qPCR was performed using nine *B. lantschouensis* heads, which included three biological replicates of the three castes. The log2 (fold-change) relative expression values obtained from the male vs. queen, male vs. worker, and worker vs. queen comparisons are shown. The black vertical lines represent error bars. In the RT-qPCR assays, the expression of the miRNAs was normalized to the U6 snRNA levels. The data were statistically analyzed using the comparative quantity (2^−ΔΔCT^) method.

Seven miRNAs identified in the male vs. queen comparison, eleven miRNAs obtained in the male vs. worker comparison, and one miRNA identified in the worker vs. queen comparison were investigated by RT-qPCR, and the results were basically consistent with the Solexa deep sequencing data (Figure 6). The male vs. queen comparison showed that seven miRNAs were upregulated, and the highest and lowest log2-fold changes in miRNA expression were 18.550773 and 1.930871, respectively. The comparison of the male vs. worker libraries revealed that 11 miRNAs were upregulated, and the highest and lowest log2-fold changes in miRNA expression were 15.93865 and 2.121722, respectively. The worker vs. queen comparison showed that one miRNA was downregulated, and the log2-fold change in miRNA expression was 0.161489.

## 4. Discussion

miRNAs are important elements that regulate gene expression and impact many vital biological functions. Specifically, miRNAs regulate the mRNA expression levels of their target genes through cleavage or translational repression in plants, mammals, and insects [6,8,9]. This study found that miRNAs were expressed at different levels in the three castes of the bumblebee *B. lantschouensis* (289 miRNAs in males, 301 miRNAs in queens, and 323 miRNAs in workers), and 171 miRNAs were common to the three castes. This finding indicated that the type of miRNAs expression among the various castes showed substantial variation and that miRNAs might have different biological functions. The analysis of the common miRNAs showed that a higher number of miRNAs was found between queens and workers than queens and males, which indicates a relationship between miRNAs and the haploid or diploid status of individuals. Our results provide valuable information for further studies on the functions of miRNAs in bumblebees.

The lengths of the miRNAs expressed in the three castes of *B. lantschouensis* were distributed between 18 and 30 nt, which is similar to the results from previous studies on queen bumblebees and honeybees [15,38]. This finding suggests that the length distribution pattern of miRNAs is conserved not only within species, but also between species. Moreover, the miRNA distribution showed a similar trend in the three castes. However, a greater number of reads with each length was found in the worker vs. queen comparison compared with the male vs. queen comparison, which indicates that these miRNAs mostly represent all of the miRNAs in the head and the same sex despite caste differences. The different read numbers might be caused by different sexes. Nonetheless, this finding should be confirmed by analyzing data from another species.

The miRNAs in the three castes were sorted by read counts with TPM normalization, and the top eight most abundant miRNAs (ame-miR-1-3p, ame-miR-276-3p, ame-miR-8-3p, ame-bantam-3p, ame-miR-2796-3p, ame-miR-317-3p, ame-miR-7-5p, and ame-miR-277-3p) in males, queens, and workers were identical, even though their rankings based on expression showed differences among the castes. These results suggest that the major miRNAs are conserved during functional regulation and play indispensable roles in bumblebee physiology. However, the expression levels of three miRNAs (ame-miR-8-3p, ame-miR-2796-3p, and ame-miR-277-3p) showed significant differences between males and queens or between males and workers, whereas no significant difference was found between workers and queens. Previous studies revealed that miRNA expression exhibits significant differences between the testes and ovaries [39,40,41]. Therefore, the expression levels of miRNAs are likely affected by caste differences.

Among the top eight most abundant miRNAs, ame-mir-2796 is reportedly involved in the development and differentiation of honeybee neurons by synergistically enhancing the role of the host gene, Phospholipase C Epsilon (PLC epsilon) and thereby regulating the division of adult workers. The miRNAs ame-mir-34 and ame-mir-317 are involved in the development of related brain functions in honeybees [42]. The miRNA bantam controls neuroblast numbers and proliferation in the central brain of *Drosophila* [43], and in honeybees, bantam, ame-miR-1-3p, ame-miR-276-3p, and ame-miR-8-3p are also highly expressed in queen and worker larvae [38,44]. Therefore, as demonstrated in previous studies, highly expressed miRNAs play important roles in the bumblebee brain.

Some of the 19 DE miRNAs identified in this study play important biological functions, as demonstrated by other authors [45,46,47,48,49,50,51,52]. For example, the let-7 family is highly expressed in gonads and plays housekeeping roles during ovarian and testicular development in insects, fishes, birds, and mammals by playing a key role in the regulation of cell proliferation and differentiation pathways [45,46,47,48,49]. Previous studies have also shown that the increase in miR-9a expression during ageing in stem and progenitor germ cells modulates degeneration in spermatogenesis and promotes detachment towards sperm maturation [50]. In this study, the expression of ame-miR-9a and ame-let-7 in males was higher than that found in females, which indicates that ame-miR-9a and ame-let-7 might play more important roles in male development. The miRNAs let-7, miR-281, and miR-125 have vital neuroprotective roles in the ageing brain, molting, and metamorphosis of insects [51,52] and might thus play different biological functions in the bumblebee head.

The putative target genes of the DE miRNAs in all three castes were enriched in 29 GO terms and 21 KEGG pathways. Catalytic activity, metabolic process, and binding were the most enriched GO terms among the three castes. The higher catalytic activity, metabolic process, and binding of males and workers might be caused by foraging activity and mating flight, which require considerable energy. Among the 21 KEGG pathways, differences between males and females were found in tryptophan metabolism and antibiotic biosynthesis pathways. Since tryptophan metabolism is vital in pregnancy, high tryptophan expression is related to queen reproduction.

The expression level of DE miRNAs measured by RT-qPCR in the three bumblebee castes was not completely consistent with the Solexa deep sequencing results. Two main reasons could account for this discrepancy: on the one hand, false positives might have occurred in the experiment, and on the other hand, low fold-change values and low relative expression of the miRNAs expressed in the three castes might be important reasons for these differences [53]. These DE miRNAs were clustered between the three bumblebee castes. Each caste was clustered in the same branch, which demonstrated that the expression of the miRNAs was specific in the different castes.

Since the genome of the Asian bumblebee, *B. lantschouensis*, has not been published, we used the genome of the European bumblebee, *B. terrestris*. We obtained high-accuracy mapping results because of the close relationship between *B. terrestris* and *B. lantschouensis* since both species belong to the same subgenus of *Bombus* s. str. The percentages of clean reads in the male, queen, and worker libraries that were mapped to the *B. terrestris* genome reached 71.61%, 69.76%, and 70.33%, respectively. Therefore, the *B. terrestris* genome can be used as a reference for providing invaluable information for high-throughput sequencing analyses of *B. lantschouensis*.

## 5. Conclusions

This study contributed new data for the identification and characterization of miRNAs in different castes of highly eusocial bees. In total, 364 known and 89 novel miRNAs were successfully identified from the small RNA libraries obtained from the heads of the three *B. lantschouensis* castes. The composition of the major miRNAs was similar in the three castes. The top eight most abundant miRNAs were found in all the castes, even though their abundances in the various castes were different, which indicated that the miRNAs that regulate different castes are relatively conserved. However, the similarity between workers and queens was higher than that between males and queens. In this study, the differences in miRNA expression between the sexes (male and female) were more pronounced than those between different reproductive states (queen and worker), even in the head. The distinct differences in behavior, physiology, longevity, and reproductive capacity between castes are driven by changes in the expression level of miRNAs. The discovery of DE miRNAs and their target genes provides a basis for understanding sex differentiation via different regulatory systems. The data obtained in this study will facilitate further research on the miRNA-mediated post-transcriptional regulation of reproduction-related genes in this species. Functional annotations and pathway analyses using the GO and KEGG databases could contribute to a better understanding of the miRNA-mediated regulation of target genes during *B. lantschouensis* development.

## Figures and Tables

**Table 1:** Forward primers used for the reverse transcription quantitative PCR (RT-qPCR) analysis of the three castes of *B. lantschouensis*.

Name	miRNA Sequence	Primer Sequence
ame-let-7-5p	UGAGGUAGUAGGUUGUAUAGU	GCGACGCTGAGGTAGTAGGTTGTATAG
ame-miR-9a-5p	UCUUUGGUUAUCUAGCUGUAUGA	GACGCTCTTTGGTTATCTAGCTGTATGA
ame-miR-31a-5p	AGGCAAGAUGUCGGCAUAGCUGA	AGGCAAGATGTCGGCATAGCTG
ame-miR-92b-3p	AAUUGCACCCGUCCCGGCCUGA	AATTGCACCCGTCCCGGC
ame-miR-100-5p	AACCCGUAGAUCCGAACUUGUG	GCAACCCGTAGATCCGAACTTGTG
ame-miR-125-5p	CCCCUGAGACCCUAACUUGUGA	GCCCCCTGAGACCCTAACTTGTG
ame-miR-263a-5p	AAUGGCACUGGAAGAAUUCACG	CAATGGCACTGGAAGAATTCACG
ame-miR-278-3p	UCGGUGGGACUUUCGUCCGUUU	TCGGTGGGACTTTCGTCCGT
ame-miR-279c-3p	UGACUAGAGUCACACUCGUCCA	GACGCTGACTAGAGTCACACTCGTCC
ame-miR-279d-3p	UGACUAGAUCCACACUCAUCCA	CGCTGACTAGATCCACACTCATCCA
ame-miR-281-3p	AAGAGAGCUAUCCAUCGACAGU	CGCTGTCATGGAGTTGCTCTCTTTG
ame-miR-3477-5p	UAAUCUCAUGCGGUAACUGUGAG	CGCTAATCTCATGCGGTAACTGTGAG
ame-miR-6001-5p	UUCUCUUUGGUUGUUACCACU	GACGCTTCTCTTTGGTTGTTACCACT
cel-miR-251	UUAAGUAGUAGUGCCGUAGAUGA	CGACGCTTAAGTAGTAGTGCCGTAGATG
dme-miR-274-5p	CUUGUGACCGUAACAACGGGCG	TTGTGACCGTAACAACGGGCG
mtr-miR2611	UAUUUGUCGAGAGUCAUUCUGA	GACGCTATTTGTCGAGAGTCATTCTGA
oni-miR-10695	UAUGUGAUCGCGGAUUUUGUC	GCTATGTGATTGCGGATTTTGTCA
str-miR-7880m-5p	UGUCGGUAGCAAAGAGGUGGUAG	GCTGTCGGTAGCAAAGAGGTGGTAG
tca-miR-3850-3p	UUCGAGACUACACGCUGAUUUU	GACGCTTCGAGACTACACGCTGATT
U6		GGCCAAGGATGACACGCAAA

**Table 2:** Classification of small RNAs belonging to different categories that are expressed in the three castes of *B. lantschouensis*.

	Male	Queen	Worker
Adapter	CTGTAGGCACCATCAAT	CTGTAGGCACCATCAAT	CTGTAGGCACCATCAAT
Raw reads	14,326,369	14,736,596	16,317,406
Clean reads	13,569,008	13,949,908	15,413,820
Mapped (%)	9,717,155 (71.61%)	9,731,221 (69.76%)	10,841,052 (70.33%)
Unmapped (%)	3,851,853 (28.39%)	4,218,687 (30.24%)	4,572,761 (29.67%)

**Table 3:** Ten most abundant miRNAs in the three castes of *B. lantschouensis*.

	Male		Queen		Worker	
Rank	miRNA	Mean Number of Reads	miRNA	Mean Number of Reads	miRNA	Mean Number of Reads
1	ame-miR-1-3p	642,846	ame-miR-1-3p	989,562	ame-miR-1-3p	963,375
2	ame-miR-276-3p	627,171	ame-miR-276-3p	650,030	ame-miR-317-3p	709,018
3	ame-miR-8-3p	573,082	ame-miR-317-3p	638,023	ame-miR-276-3p	676,149
4	ame-bantam-3p	525,099	ame-miR-277-3p	611,509	ame-miR-2796-3p	642,316
5	ame-miR-2796-3p	414,425	ame-miR-2796-3p	570,760	ame-miR-277-3p	573,485
6	ame-miR-317-3p	396,338	ame-bantam-3p	474,566	ame-bantam-3p	510,915
7	ame-miR-7-5p	372,830	ame-miR-8-3p	355,134	ame-miR-7-5p	358,513
8	ame-miR-14-3p	335,963	ame-miR-7-5p	270,861	ame-miR-8-3p	328,866
9	ame-miR-277-3p	323,674	ame-miR-184-3p	262,970	ame-miR-14-3p	280,422
10	ame-miR-9a-5p	284,686	ame-miR-34-5p	203,858	ame-miR-34-5p	277,987

**Table 4:** Differentially expressed miRNAs in the three castes of *B. lantschouensis*.

	Male vs. Queen			Male vs. Worker			Worker vs. Queen		
DEM	Log2FC	PADJ	Regulated	Log2FC	PADJ	Regulated	Log2FC	PADJ	Regulated
ame-miR-100-5p	1.03	0.004484567	Up	-	-	-	-	-	-
ame-miR-263a-5p	1.10	0.003483259	Up	1.14	0.000195153	Up	-	-	-
ame-miR-278-3p	1.32	0.000692894	Up	1.35	0.000435914	Up	-	-	-
ame-miR-279c-3p	1.69	8.42568 × 10^−15^	Up	1.41	4.02648 × 10^−08^	Up	-	-	-
ame-miR-3477-5p	1.76	3.03154 × 10^−11^	Up	1.49	3.66407 × 10^−19^	Up	-	-	-
dme-miR-274-5p	−1.33	0.003999496	Down	−2.54	3.08025 × 10^−15^	Down	1.21	0.003047672	Up
mtr-miR2611	−1.15	0.020339838	Down	-	-	-	-	-	-
oni-miR-10695	1.31	0.039998779	Up	-	-	-	-	-	-
tca-miR-3850-3p	1.19	0.026213313	Up	-	-	-	-	-	-
ame-let-7-5p	-	-	-	1.08	0.040255941	Up	-	-	-
ame-miR-125-5p	-	-	-	1.05	0.035218532	Up	-	-	-
ame-miR-279d-3p	-	-	-	1.23	2.63402 × 10^−05^	Up	-	-	-
ame-miR-31a-5p	-	-	-	1.60	0.000681818	Up	-	-	-
ame-miR-6001-5p	-	-	-	2.37	0.013708048	Up	−2.67	0.026701585	Down
ame-miR-92b-3p	-	-	-	1.01	0.027956543	Up	-	-	-
ame-miR-9a-5p	-	-	-	1.11	1.15411 × 10^−14^	Up	-	-	-
cel-miR-251	-	-	-	−1.64	2.24266 × 10^−19^	Down	1.12	2.16263 × 10^−06^	Up
str-miR-7880m-5p	-	-	-	−1.70	3.24877 × 10^−06^	Down	-	-	-
ame-miR-281-3p	-	-	-	-	-	-	1.18	8.3985 × 10^−05^	Up

DEM: differentially expressed microRNA; miRNA/miR: microRNA; FC: fold change; PADJ: *p*-value after adjustment.

## Supplementary Materials

The following are available online at https://www.mdpi.com/2075-4450/10/10/349/s1, Figure S1: Venn diagram of the distribution of known miRNAs expressed in the three castes of Apis mellifera. Figure S2: Venn diagram of the distribution of novel miRNAs in the three castes of B. lantschouensis. Table S1: Known and novel miRNAs predicted by miRDeep2.
