# Supplementary figures and images for: Analysis of miRNAs in the Heads of Different Castes of the Bumblebee Bombus lantschouensis (Hymenoptera: Apidae)
Source: Insects. 2019 Oct 16;10(10):349. doi: 10.3390/insects10100349 (PMC6835379; doi:10.3390/insects10100349)

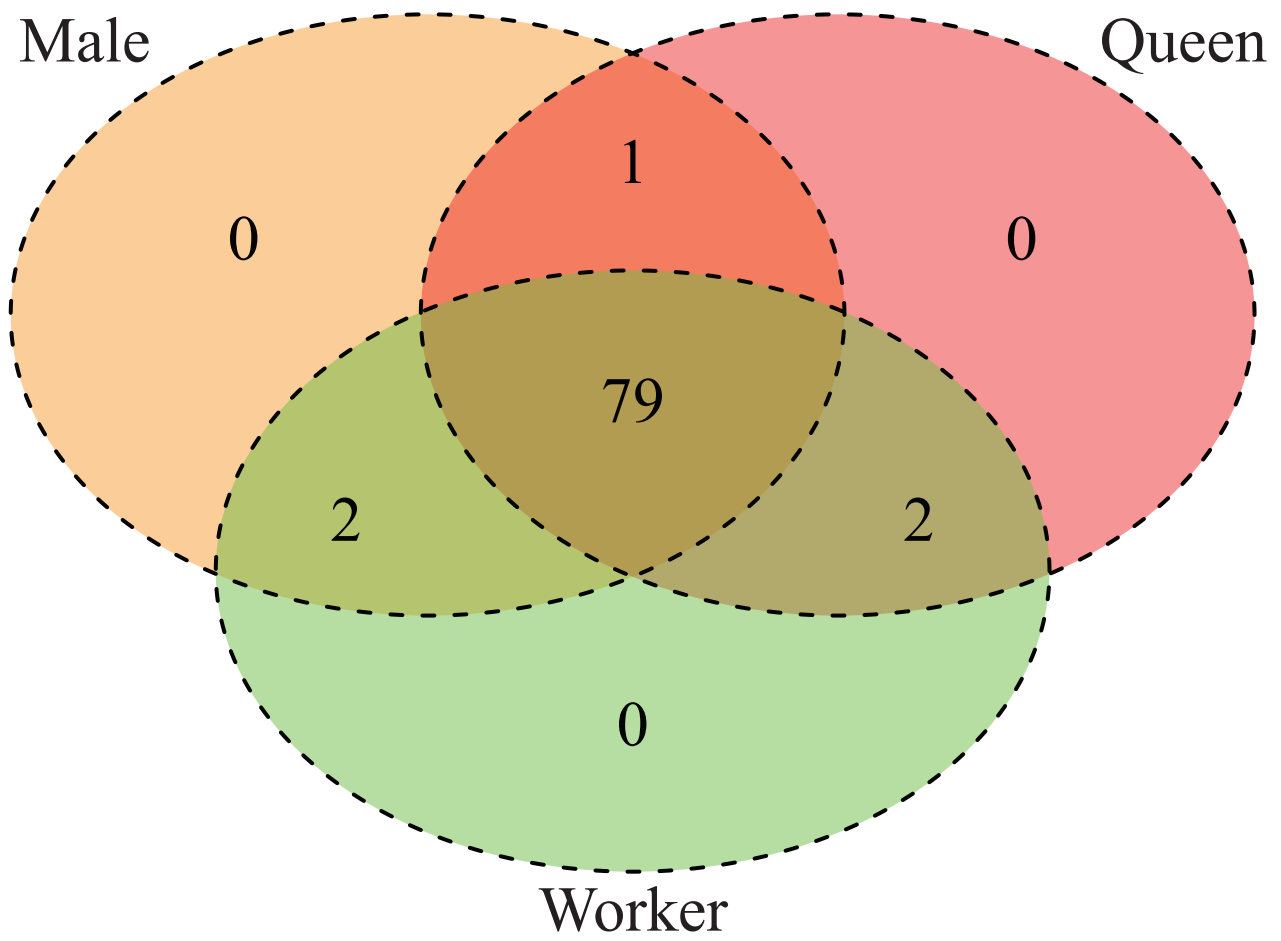

Supplement: Supplementary file 1 [file insects-10-00349-s001.zip › Appendix/Appendix/Figure A1.pdf]

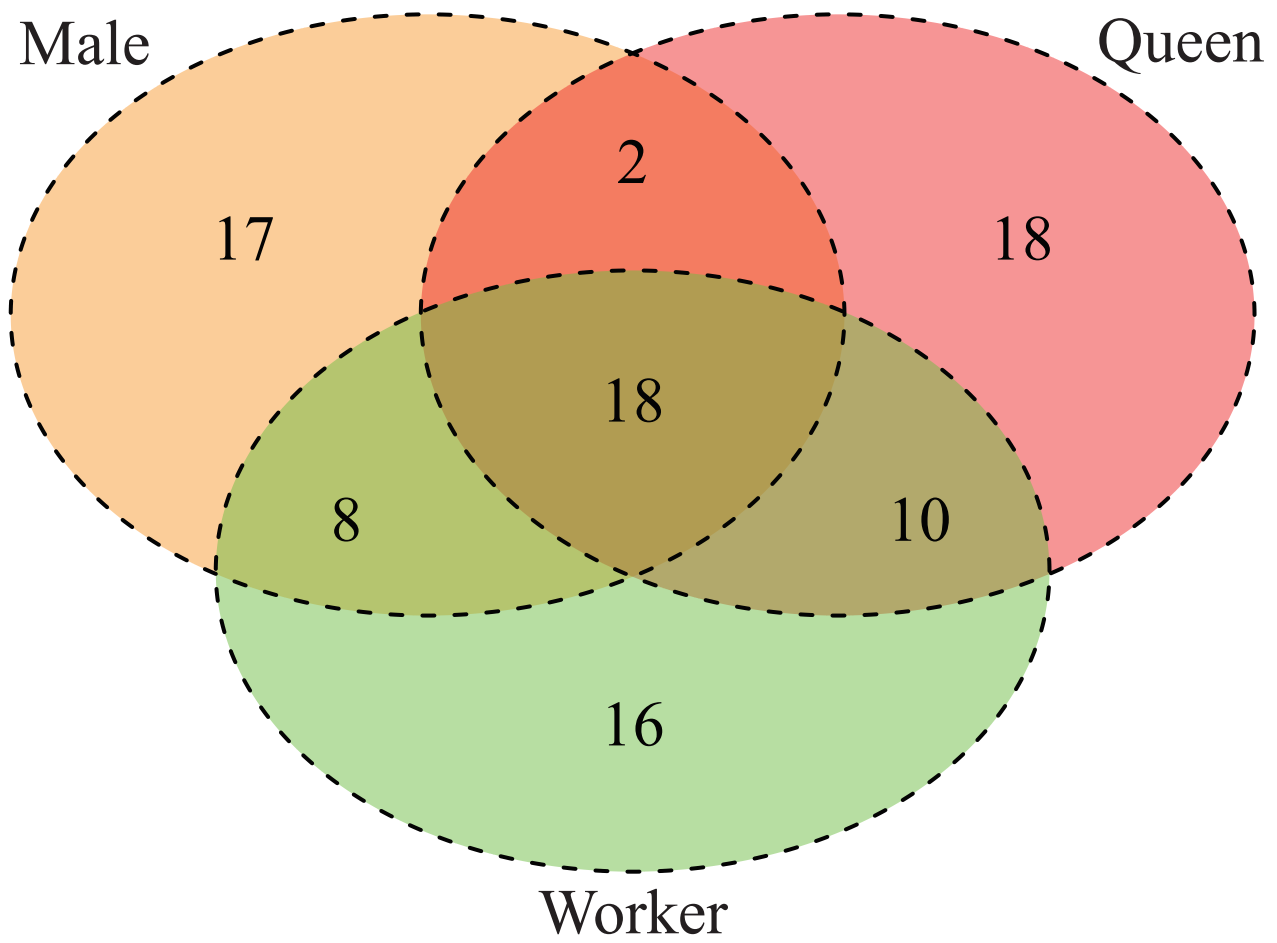

Supplement: Supplementary file 1 [file insects-10-00349-s001.zip › Appendix/Appendix/Figure A2.pdf]
